# The podium illusion: a phenomenological study of the influence of social support on well-being and performance in elite para swimmers

**DOI:** 10.1186/s13102-021-00269-1

**Published:** 2021-04-21

**Authors:** Beth Aitchison, Alison B. Rushton, Paul Martin, Andrew Soundy, Nicola R. Heneghan

**Affiliations:** 1 School of Sport, Exercise and Rehabilitation Sciences, College of Life and Environmental Sciences, University of Birmingham, Birmingham, UK; 2 The Centre of Precision Rehabilitation for Spinal Pain (CPR Spine), School of Sport, Exercise and Rehabilitation Sciences, College of Life and Environmental Sciences, University of Birmingham, Edgbaston, Birmingham, B15 2TT UK; 3 School of Physical Therapy, Western University, London, Ontario Canada; 4 English Institute of Sport, Manchester Institute of Health and Performance, Manchester, UK

**Keywords:** Para-swimming, Para-sport, Paralympic, Social support, Performance

## Abstract

**Background:**

The value of social support in enhancing performance is well established in non-impaired and sub-international level athletes with impairments. Despite this, no research to date has explored the experiences of social support in elite para-athletes. The aim of this study was to explore the experiences of social support in elite British para-swimmers and the influence on their wellbeing and performance.

**Methods:**

A hermeneutic phenomenological study involving semi-structured interviews was undertaken with 8 elite British para-swimmers (3 male, 5 female, mean age 24.9 years). Participants represented 5 para-swimming classes and all 10 of the International Paralympic Committee impairment categories. Data were analysed following a modified version of the Framework Method. Research quality and trustworthiness were ensured through employing techniques including data triangulation, member checking and reflexivity.

**Results:**

Five themes and 11 sub-themes were generated. The five themes were: ‘the coach-athlete relationship’, ‘team bond’, ‘tangible aid’, ‘The Podium Illusion’ and ‘British para-swimming’. The overall findings and the magnitude of support mentioned in the fourth theme led to the development of a new model called ‘The Podium Illusion’ which reflects the magnitude of support that is available to elite para-swimmers to help maximise their performance and wellbeing.

**Conclusion:**

Social support is essential for athlete wellbeing and performance. Findings underpin a new model, ‘The Podium Illusion’.

**Supplementary Information:**

The online version contains supplementary material available at 10.1186/s13102-021-00269-1.

## Background

The Paralympic Games is regarded as the pinnacle of para-sport and is the second largest global multi-sport event [1–4]. The profile and popularity of the Games has increased considerably since inception, with over 4000 athletes participating at the Rio 2016 Paralympic Games [5]. Para-swimming has featured at every Games, with 152 countries and 593 competitors at Rio 2016 [6]. The most successful competing nations were China, Ukraine, Great Britain (GB) and the USA, with British para-swimming the most successful GB sport [7, 8]. Elite para-athletes receive significant financial aid to support them in reaching performance targets [9–11]. Financial support to British para-swimming has increased over the years, receiving almost £11 million for the Tokyo Games [10, 11].

Despite increased awareness and investment in Paralympic sport, there is a paucity of literature on the topic of social support. Psychological research has investigated the beliefs, identity, injuries and retirement experiences elite para-athletes, [12–15] however, a notable gap is the experiences of social support. Contributing positively to athletic performance, social support comprises emotional, esteem, informational and tangible dimensions [16]. Social support is provided to non-elite para-swimmers by coaches, parents and friends, and is vital in all contexts [17]. In able-bodied athletes, social support has contributed to achieving performance targets, managing competition-associated stress and rehabilitation [18–22]. Sources of support have significantly increased over the years, with a contingency of support staff now available to athletes [23–25]. Exploration of the experiences of support in elite para-athletes is now needed; specifically British para-swimmers due to their profile and success, potentially enabling the improvement of support and performance of all para-athletes.

Based on the aforementioned considerations, the aim of the present study is to explore the experiences of social support in elite para-swimmers. Our objectives are:

- To determine the members of the elite para-swimmers’ support network.
- To explore the elite para-swimmers’ perceptions of the use of the support network and the available support.
- To explore the influence this support has on elite para-swimmer wellbeing and performance.

## Methods

See published protocol for further information on methods [26].

### Theoretical framework and study design

A qualitative, subtle-realist paradigm enabled an in-depth investigation into the para-swimmers’ experiences with social support [27]. Subtle-realism considers that knowledge is based on assumptions and human construction, and that there are multiple explanations for the same phenomena [28, 29]. Hermeneutic phenomenology was chosen to allow the exploration of the different perspectives of lived experience [27, 30–32]. For transparency, the Consolidated Criteria for Reporting Qualitative Research checklist was used for this report (Supplementary file 1) [33].

### Participants, sampling and recruitment

Eligibility criteria included: being a current British para-swimmer, an impairment which complies with the International Paralympic Committee (IPC) regulations and to have competed at the Paralympic Games or other senior international competition. Participants were purposively sampled to recruit a population who varied in their personal characteristics and experiences [34]. Varying ages, impairment categories and international competition experience enabled rich, diverse data pertinent to the study objectives [35]. Participants were recruited via email, social media and researcher networks. Sample size was based on the concept of information [36]. The recruitment rate was 100%.

The sample comprised 8 British para-swimmers (5 female, 3 male), with an age range of 18–38 years (mean = 24.9, SD = 5.74). This gender distribution is broadly representative of the British para-swimming world class pathway (WCP) [37]. Disabilities included physical, visual and intellectual, with participants competing in the swimming classes: S5, S6, S9, S12 and S14. The disabilities reported represented all 10 of the Paralympic Movement eligible impairments types. Seven participants were Paralympians and one had competed at the World Championships.

### Ethics approval and consent to participate

Ethical approval was obtained from the University of Birmingham, Ethics Committee in April 2020 (ERN_20–0344). In accordance with University policy, all procedures adhered to the research governance guidelines and regulations. All potential participants provided written informed consent.

### Procedure

Semi-structured interviews were conducted between May and June 2020 by the lead author. Due to the COVID-19 global pandemic, all the interviews were conducted via video call which still allowed for the observation of gestures and non-verbal cues. Although not an experienced researcher the lead author (conducted interviews) established participant relationships, had strong communication skills, had competed nationally in swimming for 10 years and experience of training alongside elite para-swimmers. Interview duration ranged from 48 to 88 min, (mean ± SD length 61 ± 15.3 min). The topic guide (Supplementary file 2) was based on existing research.. Participants were provided with a crib sheet, detailing definitions of social support (Supplementary file 3) [38]. Pilot and cognitive interviews were conducted with two British Paralympic athletes ahead of the main study [39, 40]. Field notes and a reflexive diary were used to aid data interpretation and increase trustworthiness [41–43].

Interviews were audio-recorded and professionally transcribed verbatim. Member checking was employed, enabling an accurate representation of participants’ experiences [41, 44].

### Data analysis

**Fig. 1:**
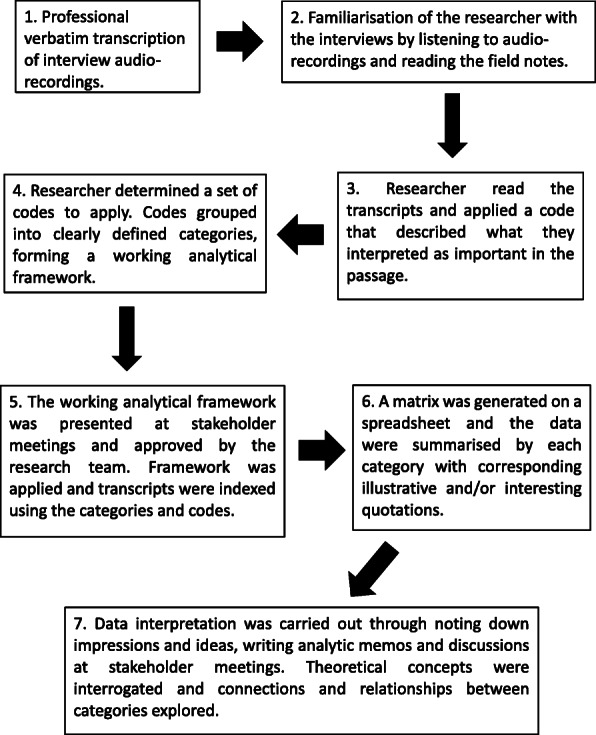
The Framework Method

The lead author was responsible for the data analysis. Following each interview, preliminary analyses, in accordance with a modified version of the *Framework Method* (Fig. 1), enabled initial interpretations and refinement of topic guide [45, 46]. Experienced qualitative researchers (AS, AR, NH) were involved in all stages of the data analysis. Microsoft Word and Microsoft Excel were used for data management.

## Results

**Fig. 2:**
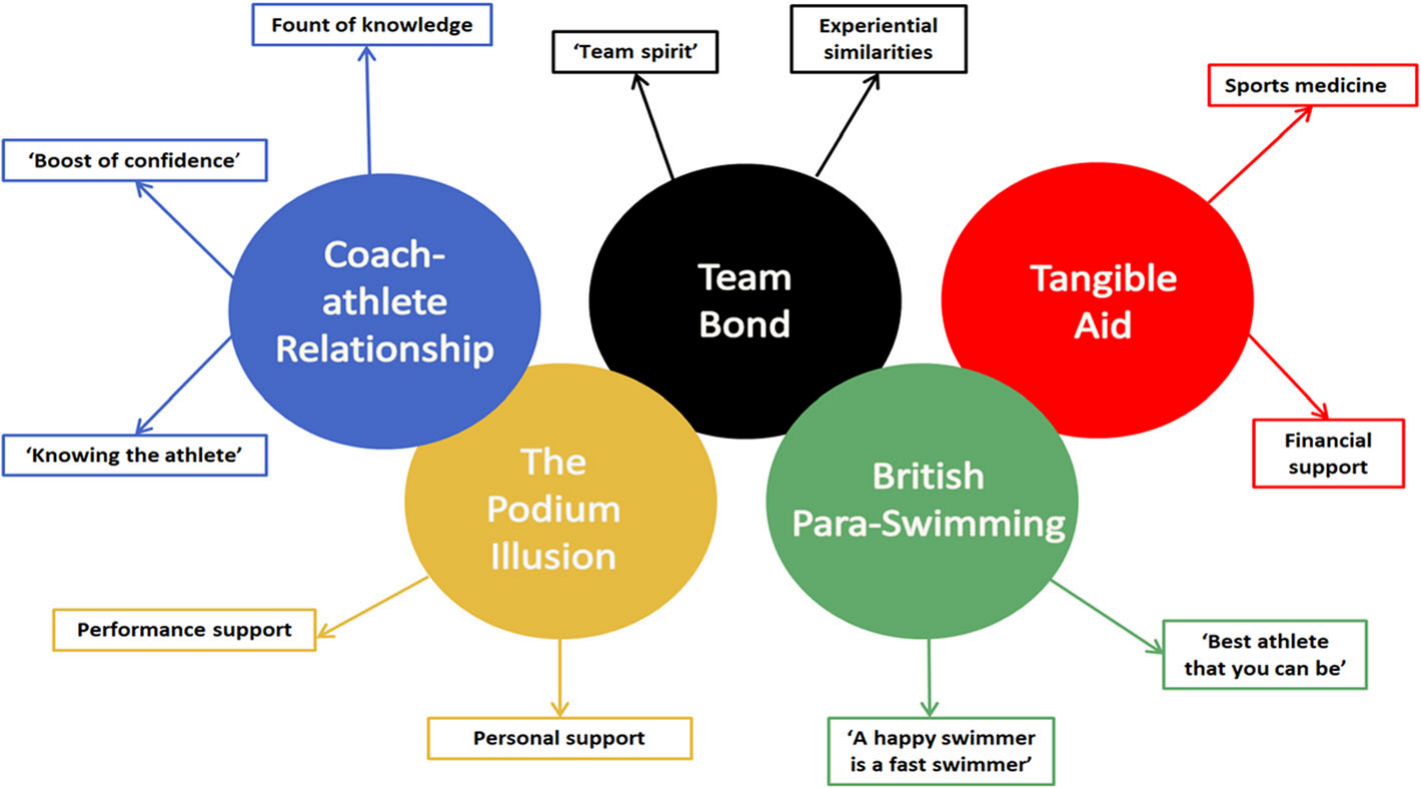
Themes and subthemes

Five themes and 11 sub-themes were generated (Fig. 2). The five themes are: coach-athlete relationship, team bond, tangible aid, British para-swimming and the Podium Illusion. The 11 subthemes are: ‘knowing the athlete’, ‘boost of confidence’, ‘fount of knowledge’, ‘team spirit’, ‘experiential similarities’, ‘sports medicine’, ‘financial support’, ‘a happy swimmer is a fast swimmer’, ‘best athlete that you can be’, ‘performance support’ and ‘personal support’. At the time, 37 para-swimmers were on the WCP, therefore limited demographic data are provided on the participants to reduce identification, following ethical considerations [37]. Supportive quotations are provided in Table 1 and Supplementary file 4.

**Table 1:** Supportive quotations for each theme and sub-theme, along with the participant (P)

Theme	Sub-theme	Quote	P
Coach-athlete relationship	‘Knowing the athlete’	*‘I can tell him [coach] absolutely everything’*	5
		*‘he [coach] was very good during the period where I wasn’t swimming, in supporting me’*	6
		*‘sometimes I don’t even know there’s something bothering me until he tells me’*	2
	‘Boost of confidence’	*‘praises you when sessions have gone well … gives you that extra boost of confidence’*	5
		*‘they [coaches] reassure me that I’ve put all the work in that I possibly could have done and then that makes me feel better’*	2
	Fount of knowledge	*‘with my pacing for my [swimming event] [asking the coach] “Do you think, if I go out a bit slower, it might help?”*’	4
		*‘Coaches have helped me structure race plans, um; what should be in my race plan, what I should be thinking about during the race. That really helps with the execution of a good race’*	6
		*‘I found out that, um, my catch was lagging for a few years, so obviously I’d just got a bit sloppy with it, um, and then we’ve [coach and participant] been working really hard on keeping my elbow high’*	2
Team bond	‘Team spirit’	*‘you’re getting up at 5:00 in the morning and you see each other at 5:00 in the morning looking horrendous, and you’ve all gotta get in the pool together’*	2
		*‘just having that kind of camaraderie and that team spirit just, kind of helps motivate you’*	4
		*‘when you’re going into a pool and your team mates have banter, like, it’s a laugh, like, it’s so much fun, they’re pushing you, you’re racing against each other, the competition, like, it makes a fun environment, like, that two hour session actually goes really fast’*	5
	Experiential similarities	*‘I’ve had a text from her [teammate] at least once a day, just saying, “Hi, what are you up to? What are you going to do?”*’	7
		*‘team-mates as well, who may have had different experiences um, along their journey, which, which can help you in a situation and, kind of, be relatable to you. I think that that can be quite useful’*	4
		*‘You can probably just stick it to the back of your mind, you know if you ever come across that experience, you’ll know how to kind of deal with it’*	2
Tangible aid	Sports medicine	*‘all helps with the recovery – physically and mentally’*	4
		*‘she’s [soft tissue therapist] the one that I just like rant to, she just listens’*	1
		*‘[physiotherapy is] just to kind of keep myself relatively loose during a heavy training period just so I don’t get too tight and trying to prevent injuries then kind of heal them once I get it’*	3
		*‘the more coaches are aware of what’s proper pre-sport, post-sport, and uh, how to kind of prevent their swimmers from getting injured, the better’*	8
	Financial support	*‘I’m really grateful for the funding because if I didn’t have it I wouldn’t be able to swim’*	2
		*‘you know like what times you’re gonna have to swim to get such and such level of funding’*	6
		*‘I wouldn’t be able to live off what I get from British swimming, there’s no way’*	7
British para-swimming	‘A happy swimmer is a fast swimmer’	*‘when I’m training well, I’m happy; but also, when I’m happy, I’m training well, if that makes sense’*	3
		*‘if you’re happy where you are and if you’ve got a good programme, obviously it’s got a good track record and good coach, um, then there’s, there’s no need to change it*’	4
		*‘I’d never move [to the NPC] because I really like that I have here’*	2
	‘Best athlete that you can be’	*‘the facilities in Manchester were amazing, the coaching was amazing, like we had everything we needed […*] *you basically couldn’t ask for a better training environment’*	6
		*‘it’d be useful if they [support staff] were a little bit closer’*	3
		*‘there’s quite a lot of support there if you want it, but you have to, you have to get it. You have to chase*	7
The Podium Illusion	Performance support	*‘The S&C (strength and conditioning) work that you do translates to the swimming work that you do’*	6
		*‘They [psychologists] gave me a lot of kind of advice, a lot of methods to help me um, when I was having a bit of a kind of plateau year’*	3
		*‘When I got back into the pool, I was then starting a different phase of training that I hadn’t done before, and what we [participant, coach and physiologist] were doing was we were monitoring how effective the gym programme had been’*	8
		*‘she’s [nutritionist] been really helpful with me trying to expand my diet and like come up with better meal plans’*	1
		*‘the biomechanics team; they’re involved in a lot of data analysis when we’re at major meets. All the swimmers will be videoed and a race report will be created post-swim um so we can go back and um, evaluate that’*	6
		*‘he’s [HPL] kind of like a bridge between us [athlete and coach] and the rest of British Swimming’*	8
	Personal support	*‘My mum and my friends keep me in the right head space to train, especially when I’m going through like injuries’*	1
		*‘I think it’s nice having a performance lifestyle advisor because they can really help you put something in place, they can help you find a bit of a career, so you never feel like, you never feel lost’*	3
		*‘He [agent] knows when I can do stuff, when I can’t due to competitions. So, again, it frees me of one less job to do, so I can actually go out there and, uh, do my training and perform, so I’m not having to think about replying to all these emails’*	5

### Theme 1: coach-athlete relationship

The coach-athlete relationship was viewed as essential and of ‘*paramount importance’* (P6), highlighting the value of having a strong relationship with a coach.

#### ‘Knowing the athlete’

There was a remarkably close relationship between the coaches and participants, with the coaches having a sense of emotional intuition when it came to their athlete. An element of trust was present between the two, with the coach providing emotional support through being approachable and a good listener. In some cases, the coach knew the participant even better than they knew themselves, reinforcing the intimate nature of the relationship.

#### ‘Boost of confidence’

Coaches praised participants during training sessions and reminded them of their hard work before races, acting as a source of esteem support. This boosted participants’ self-confidence, resulting in a perceived performance improvement. For Participant 1, her coach was able to pick up on her negativity during competitions and provide counteractive esteem support to elicit a positive performance.

#### Fount of knowledge

Coaches had a high level of technical and tactical swimming knowledge and provided informational support to the participants. They offered advice on race pacing, technique and race plans, which translated into an improvement in swimming performance for some participants.

### Theme 2: team bond

#### ‘Team spirit’

A unique bond and sense of closeness between teammates existed, which created a high level of team spirit. A sense of camaraderie and positivity made training sessions more enjoyable and pushed participants to train harder. Teammates provided esteem support, complementing participants on technique and supporting them during competitions; boosting participants’ self-confidence and having the potential to positively influence performance.

#### Experiential similarities

Due to the experiences shared, such as early morning training sessions and overseas competitions, there was a strong sense of belonging between the participants and their teammates. Teammates, both past and present, provided swimming-specific informational support to the participants, advising them on training, competitions and travelling abroad, enabling participants to learn and prepare for the future. Teammates also kept in touch with participants, checking up on them, further showing the caring nature between teammates due to the experiences shared.

### Theme 3: tangible aid

#### Sports medicine

Physiotherapists and soft tissues therapists provided tangible support through physical treatment to aid recovery, provide maintenance and reduce injury risk. The treatment received was *‘super important’* (P6), with more support sought around competitions and during heavy training periods. These individuals provided emotional support through listening and caring, and informational support through offering advice on injury prevention. Physiotherapy was also viewed as having the potential to be an educational tool if utilised correctly.

#### Financial support

Funding enabled participants to focus fully on their training and performance. Overall, the funding system was viewed as transparent and fair, although there were suggestions around increasing financial security through placing a cap on reductions after a poor season. For some, the funding they received was not sufficient and they had to seek supplementary funding support.

### Theme 4: British Para-swimming

#### ‘A happy swimmer is a fast swimmer’

The phrase ‘a happy swimmer is a fast swimmer’ is a common phrase in the swimming world. The concept being that if a swimmer is happy in themselves, their programme and with their coach, then they will swim well. Participants felt there was no need to change what was working, despite the facilities available at the semi-centralised training base – the National Performance Centre (NPC) in Manchester.

#### ‘Best athlete that you can be’

British para-swimming and the NPC were generally viewed positively, suggesting that there is a high quality of support provided, positively influencing performance. Suggestions to improve the support included maintaining communication, increasing the frequency of support, and clarifying the support available. Other issues raised were the high turnover of support staff and an occasional inability to deal with difficult situations, such as poor race performances, re-classifications, and mental health concerns.

### Theme 5: the Podium illusion

**Fig. 3:**
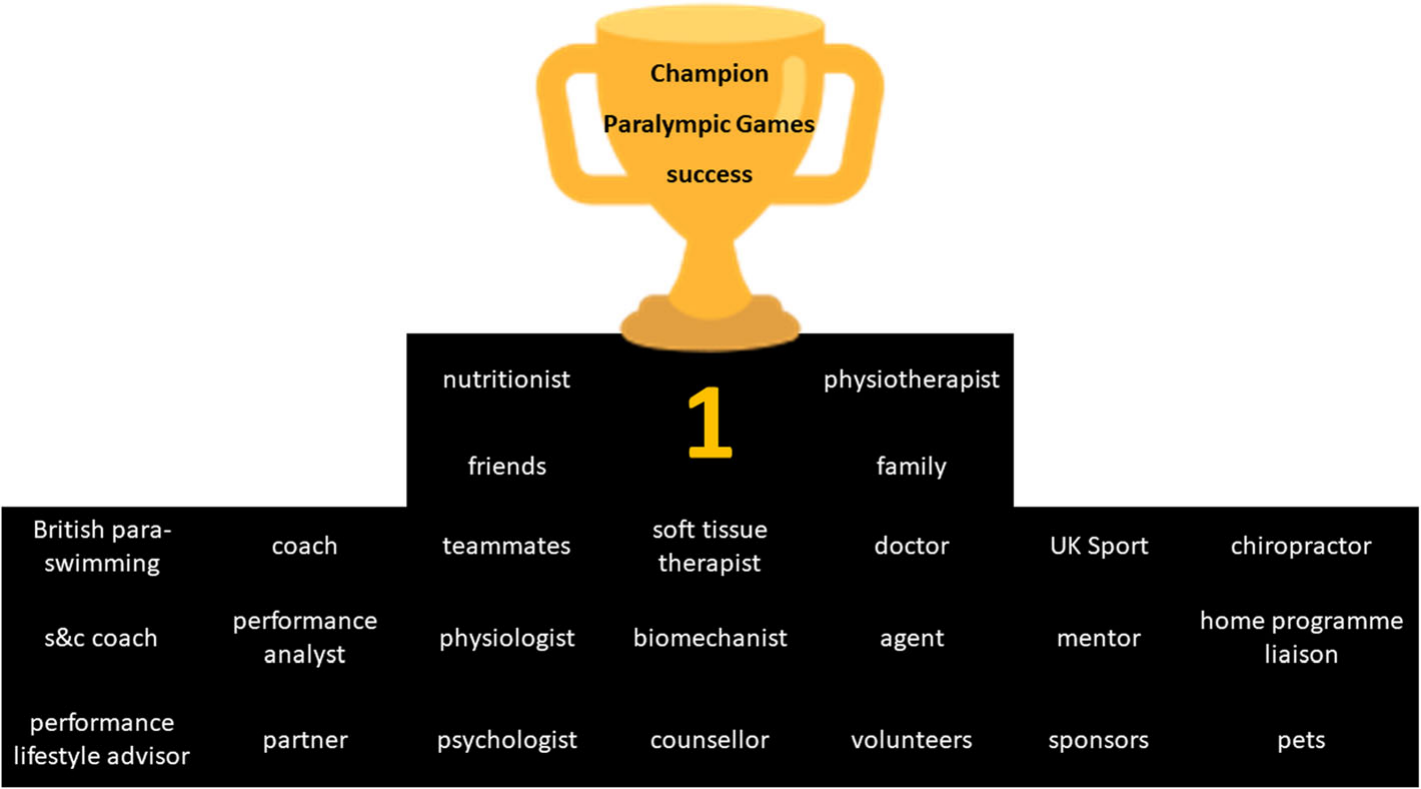
The Podium Illusion

Findings from this study resulted in the development of a new model called ‘The Podium Illusion’, which incorporates the 25 forms of support mentioned by participants (Fig. 3). It reflects how people only see an athlete’s success (i.e. the top of the podium) and not the individuals who support the athlete in achieving this success. The use of the podium makes this model potentially transferrable to all sports. This theme is split into two subthemes and explores support sources not discussed in the above themes.

#### Performance support

Several support staff were mentioned, who played a vital role in improving training and performance. These individuals included: strength and conditioning (S&C) coaches, psychologists, physiologists, nutritionists, biomechanists, race analysts and home programme liaisons.

#### Personal support

Family, friends, siblings, performance lifestyle (PL) advisors and agents also provided support, looking after participants’ mental health and overall happiness. This was associated with a perceived performance improvement through ensuring wellbeing and participants’ mental and physical capability to training.

## Discussion

This was the first study to explore elite para-swimmers’ experiences with social support and the influence it has on wellbeing and performance. Findings led to the development of a new model ‘The Podium Illusion’ that visually reflects the support available to these athletes. Key insights gained from this study suggest the importance of the coach-athlete relationship, teammates, financial aid, performance support staff and personal support from family and friends.

### Coach-athlete relationship

Coaches provided emotional, esteem and informational support. A strong coach-athlete relationship has been associated with successful performance [18, 24, 47]. The elements of closeness, respect, trust, intimacy and friendship experienced by our participants are important components of an effective coach-athlete relationship [18, 23, 24, 47, 48].

Coaches provided emotional support through being approachable, a good listener and expressing caring, which are indispensable components of the coach-athlete relationship [49]. A coach’s ability to inspire and motivate their athletes was valued, with participants experiencing increased levels of self-esteem, and improved overall wellbeing and performance with a supportive coach. This support was vital, as high confidence levels are linked with improved performance in Olympic athletes [23]. Participants received informational support which contributed to the execution of a successful race, suggesting that coaches had a good understanding of sport-specific tactical and technical elements [49].

### Team bond

A unique bond and a sense of closeness was shared between teammates, attributable to the nature of the sport and shared early morning training sessions [50]. Many studies have reported the value that teammates bring to athlete wellbeing and performance, with our participants reporting emotional, esteem and informational support.

Teammates acted as confidants and provided a sense of camaraderie, making training sessions more enjoyable. This reflects findings from other elite para-sports including cricket, sitting volleyball, badminton, wheelchair rugby and wheelchair basketball [51–56]. Advice provided by teammates was invaluable, enabling participants to maximise performance, and is reflective of existing evidence regarding managing disability and personal problem-solving [51, 53–55]. Positive remarks and complements from teammates boosted participants’ self-esteem. In contrast, negative attitudes of teammates are considered a stressor and impact performance, although not supported by our findings [57].

Support provided by teammates is particularly interesting as swimming is essentially an individual sport, with the aim being to improve individual personal best times, win medals and achieve team selections. Furthermore, team relays comprise < 5% of events at the Paralympic Games, further implying the individual nature of the sport [58]. Despite this, the impact of teammates on performance cannot be understated, suggesting that there is a squad of teammates behind every single swimmer and their individual performances.

### Tangible aid

Sports medicine support helped with recovery, maintenance and injury management, enabling both physical and mental recovery. The physiotherapist’s role is diverse, involving the provision of specific interventions to aid recovery and guidance on injury prevention [59]. Harnessing the educational power of physiotherapy to inform athletes and coaches about pre- and post-pool activities was suggested to aid injury prevention.

Funding is an integral aspect of elite para-sport, enabling participants to focus fully on their sport. Literature has suggested certain stressors that accompany the receipt of financial support, such as the expectation to perform and responsibility of national pride [57, 60, 61]. However no participants recalled any funding-associated stressors, possibly due to security in funding status due to their high global rankings. Some sought additional financial support to supplement finances and cover living costs, which has been the case for other previous British para-athletes [61]. A standardised amount of funding may not be sufficient to meet the specific individual athlete’s needs.

### British Para-swimming

Participants were appreciative of the many facilities available at the NPC, such as the Kistler Performance Analysis System to analyse starts, turns and relay takeovers [62–64]. Despite this, many were happy in their home programmes, suggesting that the coach and training environment are more important. Some participants thought their home club location negatively influenced the frequency of contact with support staff. If the support and resources were more accessible then participants may seek this more frequently, potentially resulting in an improved performance.

To create more stability, staff retention should be reviewed. The need for good communication was highlighted, keeping athletes up to date to remove any unnecessary stressors [65]. The lack of support from support staff in certain situations, such as following poor race performances, was also raised. Elite para-athletes can experience both sport and disability-specific stressors which can affect wellbeing and performance, with the process of re-classification being particularly traumatic and isolating, further demonstrating the need for appropriate mental health support from British para-swimming staff [2, 14, 66–68].

### The Podium illusion

Study findings led to the development of a new model ‘The Podium Illusion’ which reflects the immensity of support provided to elite para-swimmers and the influence on their performance and wellbeing (Fig. 3). This model is potentially transferrable to all sports as all elite athletes receive at least some of the forms of support depicted in this model. It was apparent that more support was sought around competition times, for example to prime the body with S&C related activities before races, to measure race-specific parameters and to receive race day nutritional advice. This suggests that the support provided by practitioners to para-swimmers should be greater and more readily available around competition periods.

Strength and conditioning coaches prescribed swimming-specific on-land exercises, which directly translated into perceived swimming improvements, and is linked to enhanced performance and injury reduction [69–73]. Participants sought more psychology support when overcoming mental challenges, such as when experiencing post-Paralympic blues or a plateau year [74–76]. Support provided by psychologists can help athletes prepare mentally for the demands of elite sport, and has been shown to improve swimming performance [77]. Physiologists conducted tests and measured swimming specific parameters (e.g. heart rate, stroke rate, lactate levels), which enables the provision of advice to inform training [78]. Nutritionists provided informational support, giving advice on diets, fuelling for training sessions and race day nutrition, ensuring an effective performance, and reducing injury and illness risk [79]. Skinfold measurements taken by nutritionists may provide insight into optimal body composition specific to each athlete, maximising performance [80]. Performance analysis enabled the evaluation and improvement of technique, pacing and specific race components, and has been found to improve Paralympic swimming performance through race strategy adjustment [81, 82].

Personal support was viewed as vital to success and happiness. Family and friends provided emotional support to participants, who experienced a sense of closeness, trust and understanding. Emotional support is vital to help athletes cope with stressors, recover from injury and aid retirement from sport [24, 83, 84]. PL advisors mentored participants to facilitate their personal development and plan for life after swimming. This role is important in ensuring the balance between athlete wellbeing and the pursuit of high-performance sport [85, 86].

### Strengths and weaknesses

The sample was representative of British para-swimming allowing an in-depth exploration of social support [34, 35]. Measures were undertaken to optimise rigour including reflexivity, triangulation and member checking, ensuring trustworthiness of the results [44, 87, 88]. Finally, the research team comprised experienced qualitative researchers and an elite-para sport practitioner, aiding the interpretation and analysis process.

If method triangulation had been employed (i.e. observations), there may have been a deeper understanding of social support experiences [89, 90]. Secondly, there was no variation in participant nationality or ethnicity meaning that the findings are only applicable to white British athletes. Thirdly, the *Framework Method* states that an experienced qualitative researcher should ideally lead the analysis and be involved at every stage [46]. In our case the study was led by a relatively novice researcher although oversight and active participation from those with considerable methodological and subject-specific expertise was ensured throughout.

### Implications for practice

Findings suggest that a whole contingency of specialised, knowledgeable support staff contributes substantially to athletic success due to the range of social support provided covering all aspects of performance. The new model created, ‘The Podium Illusion’, is potentially transferable to all elite sports and visually reflects the support an elite athlete receives. More support was sought around competitions, suggesting that the support offered should be increased before, during and post-competition to reflect this.

The coach-athlete relationship is an essential component of elite sport and an athlete should seek a coach with whom they can form a close, trusting relationship. Teammates provided camaraderie and motivational support, suggesting they also are vital to success. The high-level performances would not have been possible without funding and the subsequent impact of reduced funding on performance outcomes should be considered by a National Governing Body (NGB). Personal support was also of vital importance, implying that an athlete is unlikely to perform to such high levels without good overall wellbeing. The suggestions made for British para-swimming could be considered by other NGBs should they desire to improve the support provided.

### Further research

Further research is needed to build on these findings and provide a greater depth of knowledge in this field. This phenomenon should be explored in elite para-swimming populations in other countries, allowing the comparison of the availability and quality of social support across swimming nations. The experiences of social support should also be explored in other para-sports within GB to investigate and compare the support available to athletes between sports. These explorations and comparisons would enable identification of the areas for improvement in specific sports and allow sports to build on their successes, maximising the performance and wellbeing of their athletes. Future studies could also focus specifically on the two different types of support available to athletes: performance and personal, allowing a more in-depth investigation into the role of these forms of support on athlete success and wellbeing. Studies could also consider specifically the role of financial support as it appears to be multi-faceted, with athletes receiving support from funding, sponsorship, donations and family.

## Conclusion

Findings evidence the importance of social support in British para-swimmers for performance and wellbeing, notably a strong coach-athlete relationship, motivational and supportive teammates, and sufficient financial and NGB backing. The sheer volume of support available to elite para-athletes is reflected in the new model: ‘The Podium Illusion’, which highlights the performance and personal support received by the para-swimmers. Findings have the potential to inform future advances in the quality and quantity of support received, maximising athlete wellbeing and performance.

## Supplementary Information

**Additional file 1.**

**Additional file 2.**

**Additional file 3.**

**Additional file 4.**

## Data Availability

The datasets used and/or analysed during the current study available from the corresponding author on reasonable request.

## Abbreviations

GB: Great Britain
IPC: International Paralympic Committee
NGB: National Governing Body
NPC: National Performance Centre
S&C: Strength and conditioning
WCP: World class pathway
