# Supplementary material for: The podium illusion: a phenomenological study of the influence of social support on well-being and performance in elite para swimmers
Source: BMC Sports Sci Med Rehabil. 2021 Apr 21;13:42. doi: 10.1186/s13102-021-00269-1 (PMC8058746; doi:10.1186/s13102-021-00269-1)
Supplement: Supplementary file 1 — Additional file 1. [file 13102_2021_269_MOESM1_ESM.pdf]

The Podium Illusion: A phenomenological study of the influence of social support on well-being and performance in elite para swimmers

Authors Beth Aitchison, Alison B Rushton, Paul Martin, Andrew Soundy, Nicola R Heneghan

No. Item	Guide questions	Description	Location in manuscript
Domain 1: research team and reflexivity			
Personal characteristics			
1. Interviewer/facilitator	Which author/s conducted the interview or focus group?	BA	Page 6 Procedure
2. Credentials	What were the researcher's credentials? E.g. PhD, MD	MSc student, experienced researchers and para-sport practitioner.	Page 19 Authors contributions
3. Occupations	What was their occupation at the time of the study?	MSc by research student	Page 19 Authors contributions
4. Gender	Was the researcher male or female?	Female	Page 19 Authors contributions
5. Experience and training	What experience or training did the researcher have?	No formal training in interviewing but the researcher had strong communication skills and had undertaken pilot and cognitive interviews.	Page 6 Procedure
Relationship with participants			
6. Established relationship	Was a relationship established prior to study commencement?	Yes the researcher conversed with participants prior to the interview and developed rapport.	Page 6 Procedure
7. Participant knowledge of the interviewer	What did the participants know about the researcher? e.g. personal goals, reasons for doing the research	Participants were informed of the purpose of the study and that it formed part of the researcher's MSc.	N/A (protocol)
8. Interviewer characteristics	What characteristics were reported about the inter viewer/facilitator? e.g. Bias, assumptions, reasons and interests in the research topic	Interviewer characteristics and interests were reported.	Page 6 Procedure Page 19 Author contributions
Domain 2: study design			
Theoretical framework			

The Podium Illusion: A phenomenological study of the influence of social support on well-being and performance in elite para swimmers

Authors Beth Aitchison, Alison B Rushton, Paul Martin, Andrew Soundy, Nicola R Heneghan

9. Methodological orientation and theory	What methodological orientation was stated to underpin the study? e.g. grounded theory, discourse analysis, ethnography, phenomenology, content analysis	Hermeneutic phenomenology	Page 5 Theoretical framework and study design
Participant selection			
10. Sampling	How were participants selected? e.g. purposive, convenience, consecutive, snowball	Purposive sampling	Page 5 Participants, sampling and recruitment
11. Method of approach	How were participants approached? e.g. face-to-face, telephone, mail, email	Email, social media and researchers' contacts.	Page 5 Participants, sampling and recruitment
12. Sample size	How many participants were in the study?	Eight	Page 7 Results
13. Non-participation	How many people refused to participate or dropped out? Reasons?	Eight participants were invited to participant and all gave informed consent and completed the interview. No participants refused participation or dropped out.	Page 5 Participants, sampling and recruitment
Setting			
14. Setting of data collection	Where was the data collected? e.g. home, clinic, workplace	Interviews were conducted over video call e.g. skype and zoom.	Page 6 Procedure
15. Presence of non-participants	Was anyone else present besides the participants and researchers?	One additional person was present in two interviews; however their comments were discarded and not included in the analysis.	N/A
16. Description of sample	What are the important characteristics of the sample? e.g. demographic data, date	The sample comprised five females and 3 males aged 18-38 years. They represented five swimming classifications and had varying international competition experience. Data was collected between May-June 2020.	Page 7 Results
Data collection			
17. Interview guide	Were questions, prompts,	A topic guide was used to	Page 6

	guides provided by the authors? Was it pilot tested?	conduct the interviews. It was informed by literature and discussions with para-sport practitioners about the social support available. Pilot and cognitive interviews were conducted with two Paralympians prior to the study.	Procedure Supplementary file 3
18. Repeat interviews	Were repeat interviews carried out? If yes, how many?	No.	N/A
19. Audio/visual recording	Did the research use audio or visual recording to collect the data?	Interviews were audio-recorded.	Page 6 Procedure
20. Field notes	Were field notes made during and/or after the interview or focus group?	Field notes were made during the interview when necessary.	Page 6 Procedure
21. Duration	What was the duration of the interviews or focus group?	48-88 minutes.	Page 6 Procedure
22. Data saturation	Was data saturation discussed?	No	N/A
23. Transcripts returned	Were transcripts returned to participants for comment and/or correction?	Yes member checking was carried out.	Page 6 Procedure
Domain 3: analysis and findings			
Data analysis			
24. Number of data coders	How many data coders coded the data?	One – BA.	Page 6 Data analysis
25. Description of the coding tree	Did authors provide a description of the coding tree?	No.	N/A
26. Derivation of themes	Were themes identified in advance or derived from the data?	Themes were derived from the data.	Page 6 Data analysis
27. Software	What software, if applicable, was used to manage the data?	Microsoft word and Microsoft excel.	Page 6 Data analysis
28. Participant checking	Did participants provide feedback on the findings?	No.	N/A
Reporting			
29. Quotations	Were participant quotations	Yes comments made were	Table 2.

presented	presented to illustrate the themes/findings? Was each quotation identified? e.g. participant number	supported by quotations from the participants. Each quotation was identified using a participant number.<br>Additional quotations are present in supplementary file 6.	Supplementary file 4.
30. Data and findings consistent	Was there consistency between the data presented and the findings?	Yes.	Page 8-13 Results
31. Clarity of major themes	Were major themes clearly presented in the findings?	Yes.	Page 8-13 Results
32. Clarity of minor themes	Is there a description of diverse cases or discussion of minor themes?	Yes.	Page 8-13 Results
