# Supplementary material for: The podium illusion: a phenomenological study of the influence of social support on well-being and performance in elite para swimmers
Source: BMC Sports Sci Med Rehabil. 2021 Apr 21;13:42. doi: 10.1186/s13102-021-00269-1 (PMC8058746; doi:10.1186/s13102-021-00269-1)
Supplement: Supplementary file 2 — Additional file 2. [file 13102_2021_269_MOESM2_ESM.pdf]

	**The Podium Illusion: A phenomenological study of the influence of social support on well-being and performance in elite para swimmers**<br><br>**Authors** Beth Aitchison, Alison B Rushton, Paul Martin, Andrew Soundy, Nicola R Heneghan

Interview Section	Questions/Content	Prompts	Aims
Ethics Statement	Firstly, I would like to thank you for participating in this interview. Just a reminder that it will be audio-recorded but all information shared will be kept strictly confidential. You are entitled to stop the interview and the recording at any point or terminate the interview altogether if you wish.<br><br>You also have the right not to answer a question if you do not wish to. There are no right or wrong answers. I am interested in your own personal experiences, thoughts and perceptions, with the aim of today being to understand your experiences of being an elite para-athlete and your support network.<br><br>Before we start do you have any further questions?	• *Can I confirm that you have read and understand the information sheet and signed the consent form?*<br>• *Are you comfortable?*	• To ensure full understanding of what is expected of the participant during this interview.<br>• Make sure the participant is comfortable and ready to begin.
	We're going to start with a few introductory questions so that I can get to know you a little more. This section will just take a few minutes.		
Introductory Questions	1. Can you tell me a bit about yourself?<br><br>2. Can you tell me about your sporting background?<br><br>3. Can you tell me about your classification, impairment and main event/s?<br><br>4. Can you tell me about your previous Paralympic Games/International competition experiences?	• *Age, where you come from, studying, job, personality. What 3 words or phrases would you use to describe yourself?*<br>• *When did you start? How you became involved? What made you start? What support did you have getting involved in swimming? Were there any barriers to getting involved? Why do you swim? Your achievements?*<br>• *What are you best swimming events? What classification? What impairment? How does it affect you day to day? Use of any aids or prosthesis. In short, how do you feel about the current classification system?*<br>• *What Games/International competitions have you been to? What support did you receive in getting there, during and after?*	• Make participant relax and feel comfortable with talking and opening up.<br>• Build rapport.<br>• To gain an insight into the participant's background and their sporting career.

		Positives/negatives/successes. Have you found this experience has changed across the years? (If been to more than one).	
Transition Questions	1. What is your overall experience of being an elite swimmer?<br><br>2. What does you daily/weekly training schedule look like? Can you run me through it?	• *What does being a swimmer mean to you?*<br>• *What aspects of being an athlete do you enjoy?*<br>• *Any challenges with being an elite athlete?*<br>• *Benefits/positives/negatives*<br>• *Best experience and worst experience? (If struggling to answer)*<br>• *Have you seen any changes in the support available to swimmers change over time?*<br><br>• *How many times a week do you train? For how long? Any land based training?*<br>• *How frequently do you have competitions?*<br>• *Do you go on any training camps?*<br>• *Who goes with you to competitions and training camps in terms of support staff?*<br>• *What support do you have access to as a GB funded swimmer?*<br>• *What do you like to do in your spare time to relax and de-stress?*	• Start to guide the interview towards experiences of being a Paralympic athlete.<br>• To get an idea of training schedule.<br>• May prompt athlete to bring up support staff (eg. Seeing physiotherapist, psychologist, sports massage).
	To start with, earlier you mentioned X (support staff) who you have contact with as part of your social network. Could you tell me a bit about their role and the support they give you?	• *How often do you see this person/use this support?*<br>• *In what way do they support/help you? In terms of performance? In terms of wellbeing?*<br>• *Do you feel there are any benefits to seeing this person?*<br>• *Are there any negatives associated with seeing this person? Any negative experiences?*<br>• *What influence, if any, do other people have on your seeking of this support?*<br>• *Do you access different support/go to different people for support in different situations?*<br>• *Who else supports you in achieving your performance goals?*<br>• *What role do teammates play in support?*	• To determine who provides (structural) support and the members of the social network.<br>• To determine the benefits that each person provides to the participant's wellbeing and performance.<br>• Then move on to the next person mentioned by the athlete.
Social support background	We will now move on to the main research questions focused on social support. Many studies have demonstrated the importance of social support in maintaining and improving physical and psychological health.	• *Do you have any questions?*<br>• *If you are unsure at any point regarding the types of support, please ask.*	• Inform the participant of the background of the study<br>• Ensure the participant knows they can ask

	Social support is split into functional support and structural support. There are 4 different types of functional support: emotional, esteem, informational and tangible. Structural support is concerned with your support network and who you talk to and see on a regular basis.<br><br>These types of support can be provided by different people. They could include family, close friends, partner, teammates, support staff and coaches.<br><br>Before each question I will define what is meant by each type of support. If you are not clear on what is meant, then please let me know.	• *Use the social support definitions sheet provided to guide you if needed.*	questions if they are unsure.
Main questions	1. Emotional support is defined as 'the ability to turn to others for comfort and security during times of stress, leading the person to feel that he or she is cared for by others'.<br><br>Is there anyone who provides this type of support to you?	• *In what way do they provide support?*<br>• *Can you tell me how important is this form of support to you?*<br>• *How often do you use this support?*<br>• *Why do you use/need this support?*<br>• *In what way does this support benefit your performance? What about wellbeing?*<br>• *Are there situations where you access this support more? Why?*<br>• *Does your impairment and/or heath influence your seeking of this support?*<br>• *What influence, if any, do other people have on your seeking of this support?*<br>• *Have you had any negative experiences with this form of support? Can you give an example of this?*<br>• *Is there any way this support could be improved?*	• To determine who provides emotional support to the participant.<br>• To determine how important emotional support is.<br>• To determine in what way emotional support benefits performance and wellbeing.<br>• To determine the situations and factors that influence the seeking of emotional support.
	2. Esteem support is 'the bolstering to a person's sense of competence or self-esteem by other people. Giving an individual positive feedback on his or her skills and abilities or expressing a belief that the person if capable of coping with a stressful event'.	• *In what way do they provide support?*<br>• *Can you tell me how important is this form of support to you?*<br>• *How often do you use this support?*<br>• *Why do you use/need this support?*<br>• *In what way does this support benefit your performance? What about wellbeing?*<br>• *Are there situations where you access this support more? Why?*<br>• *Does your impairment and/or heath influence your seeking of this support?*	• To determine who provides esteem support to the participant.<br>• To determine how important esteem support is.<br>• To determine in what way esteem support benefits performance and wellbeing.<br>• To determine the situations and factors that influence

	Is there anyone who provides this type of support to you?	• *What influence, if any, do other people have on your seeking of this support?*<br>• *Have you had any negative experiences with this form of support? Can you give an example of this?*<br>• *Is there any way this support could be improved?*	the seeking of esteem support.
	3. Informational support is 'providing the individual with advice or guidance concerning possible solutions to a problem'.<br><br>Is there anyone who provides this type of support to you?	• *In what way do they provide support?*<br>• *Can you tell me how important is this form of support to you?*<br>• *How often do you use this support?*<br>• *Why do you use/need this support?*<br>• *In what way does this support benefit your performance? What about wellbeing?*<br>• *Are there situations where you access this support more? Why?*<br>• *Does your impairment and/or heath influence your seeking of this support?*<br>• *What influence, if any, do other people have on your seeking of this support?*<br>• *Have you had any negative experiences with this form of support? Can you give an example of this?*<br>• *Is there any way this support could be improved?*	• To determine who provides informational support to the participant.<br>• To determine how important informational support is.<br>• To determine in what way informational support benefits performance and wellbeing.<br>• To determine the situations and factors that influence the seeking of informational support.
	4. Tangible support is 'concrete instrumental assistance in which a person in a stressful situation is given the necessary resources to cope with the stressful event' (eg. Financial assistance, physical help).<br><br>Is there anyone who provides this type of support to you?	• *In what way do they provide support?*<br>• *Can you tell me how important is this form of support to you?*<br>• *How often do you use this support?*<br>• *Why do you use/need this support?*<br>• *In what way does this support benefit your performance? What about wellbeing?*<br>• *Are there situations where you access this support more? Why?*<br>• *Does your impairment and/or heath influence your seeking of this support?*<br>• *What influence, if any, do other people have on your seeking of this support?*<br>• *Have you had any negative experiences with this form of support? Can you give an example of this?*<br>• *Is there any way this support could be improved?*<br>• *What are your thoughts on the funding available to you and what you receive?*	• To determine who provides tangible support to the participant.<br>• To determine how important tangible support is.<br>• To determine in what way tangible support benefits performance and wellbeing.<br>• To determine the situations and factors that influence the seeking of tangible support.
	Is there anything else around your support network or experiences of support that we haven't covered which you would like to add or you think is of relevance?		

Conclusion	That’s all the questions I have so the interview has now finished. Thank you for participating in this study, I really appreciate your time and input.	• *Is there anything you would like to ask regarding the analysis of the data or the next steps of the process?*<br>• *Ensure participant does not need signposting towards any wellbeing services, do so if necessary.*	• Ensure the participant is comfortable with what has been discussed.
