# Supplementary material for: The podium illusion: a phenomenological study of the influence of social support on well-being and performance in elite para swimmers
Source: BMC Sports Sci Med Rehabil. 2021 Apr 21;13:42. doi: 10.1186/s13102-021-00269-1 (PMC8058746; doi:10.1186/s13102-021-00269-1)
Supplement: Supplementary file 3 — Additional file 3. [file 13102_2021_269_MOESM3_ESM.pdf]

The Podium Illusion: A phenomenological study of the influence of social support on well-being and performance in elite para swimmers

Authors Beth Aitchison, Alison B Rushton, Paul Martin, Andrew Soundy, Nicola R Heneghan

Social Support Definitions

Taken from the research of Cutrona and Russell (1990).

The below are just examples - they are not a definitive list of social support providers or services.

Emotional support

- Support given to provide comfort to an individual for them to feel that they are cared for and can feel secure at times of stress.
- Eg. Support from anyone close to you in the form of listening, comfort, encouragement and demonstrations of caring and love.

Esteem support

- Support that helps bolster or encourage an individual's self-esteem or sense of competence. Provisionally through providing positive feedback on an individual's skills or abilities and expressing that an individual is capable of achieving and coping with a stressful event or task.
- Eg. Feedback from coach about training, performance or technique. Support of family, friends, teammates or others. Support designed to improve your self-esteem and confidence.

Informational support

- Support that provides a person with advice or guidance concerning possible solutions to a problem.
- Eg. Advice from coaches and support staff to improve training and performance. Talking to teammates with experiential similarities. Guidance or feedback from family, friends or others.

Tangible support

- Support that provides some instrumental or concrete assistance to an individual.
- Eg. Seeing and working with support staff for physical treatment or training. Receiving performance-related assistance from support staff, such as nutritional and psychological. Receiving financial aid.
