# Supplementary material for: The podium illusion: a phenomenological study of the influence of social support on well-being and performance in elite para swimmers
Source: BMC Sports Sci Med Rehabil. 2021 Apr 21;13:42. doi: 10.1186/s13102-021-00269-1 (PMC8058746; doi:10.1186/s13102-021-00269-1)
Supplement: Supplementary file 4 — Additional file 4. [file 13102_2021_269_MOESM4_ESM.pdf]

The Podium Illusion: A phenomenological study of the influence of social support on well-being and performance in elite para swimmers

Authors Beth Aitchison, Alison B Rushton, Paul Martin, Andrew Soundy, Nicola R Heneghan

Theme	Sub-theme	Quotation	P
**The coach-athlete relationship**	**'Knowing the athlete'**	'she [coach] understands'	1
		'I can tell him [coach] absolutely everything'	5
		'he [coach] understands me. I'm not just an athlete. I'm not a robot who can swim top sessions all the time […] Like, I'm a human being as well, like, I'm a girl, like, I, I have shitty moods when I'm on periods and stuff, and he's, like, he's, he's so understanding of that'	5
		'he [coach] can help me talk through it [problems] or sort it out'	8
		'I can have a good cry with my coach and then I just feel like a million times better after because it feels like a weight has been lifted off your shoulders'	2
		'My coach is probably a good listener and he kind of gets rid of all my worries, 'cause my worries are normally around the sport'	2
		'he [coach] was very good during the period where I wasn't swimming, in supporting me'	6
		'there was a time where I was, like, so emotional we just stopped the session and we [coach and participant] just talked'	5
		'sometimes he'll pick up that I'm not right and then he'll call me for a chat'	2
		'sometimes I don't even know there's something bothering me until he tells me'	2
		'I'm super close to my coach'	5
	**'Boost of confidence'**	'there might be times where you need somebody just to, kind of, big you up a little bit'	4
		'praises you when sessions have gone well… gives you that extra boost of confidence	5
		'"Oh, that was a good training session. That will help you in however many months"'	3
		'they [coaches] reassure me that I've put all the work in that I possibly could have done and then that makes me feel better'	2
	**Fount of knowledge**	'All the stuff that's swimming related they just like feed it back to you like. There's a lot, there's a lot under that umbrella.'	6
		'with my pacing for my [swimming event] [asking coach] "Do you think, if I go out a bit slower, it might help?"'	4
		'I felt like I was a bit heavy on my right arm, I could say "Oh, does it look like it's heavy?"'	4
		'Coaches have helped me structure race plans, um; what should be in my race plan, what I should be thinking about during the race. That really helps with the execution of a good race'	6
		'I found out that, um, my catch was lagging for a few years, so obviously I'd just got a bit sloppy with it, um, and then we've	2

		[coach and participant] been working really hard on keeping my elbow high’	
**Team bond**	**‘Team spirit’**	‘you’re getting up at 5:00 in the morning and you see each other at 5:00 in the morning looking horrendous, and you’ve all gotta get in the pool together’	2
		‘just having that kind of camaraderie and that team spirit just, kind of helps motivate you	4
		‘when you’re going into a pool and your team mates have banter, like, it’s a laugh, like, it’s so much fun, they’re pushing you, you’re racing against each other, the competition, like, it makes a fun environment, like, that two hour session actually goes really fast’	5
		‘sometimes they [teammates] say little comments, remarks to pick you up and get you going again’	2
		‘when it comes to competitions […] if you’ve got a good relationship with your, your teammates, um, you, you can kind of trust that they are gonna want the best for you’	4
		‘they [teammates] say “Oh, you looked good, (name of participant), in the water” […] little things like that that, again, it gives you that big confidence booster and that positivity’	5
		‘they [teammates] definitely push you on as well, especially, sometimes if you see someone else doing well it makes you do well’	2
		‘I really like having a lot of people [teammates] because you have so many different personalities and I think when you have a lot of people, say if there’s a few people missing, you still kind of have enough people to kind of really bounce off’	3
		‘I have had other swimmers tell me that I’m shit but that’s just them trying to psych you out. […] it normally makes me swim harder, to be fair because I’m annoyed’	2
	**Experiential similarities**	‘you’re all in this together’	2
		‘your teammates are your closest friends too. You see them morning and evening’	5
		‘I’ve had a text from her [teammate] at least once a day, just saying, “Hi, what are you up to? What are you going to do?”’	7
		‘[talking to] team-mates as well, who may have had different experiences um, along their journey, which, which can help you in a situation and, kind of, be relatable to you. I think that that can be quite useful.’	4
		‘You can probably just stick it to the back of your mind, you know if you ever come across that experience, you’ll know how to kind of deal with it’	2
		‘[talking to ex-swimmers] makes you sometimes step away from that bubble’	5
**Tangible aid**		‘all helps with the recovery – physically and mentally’	4

The Podium Illusion: A phenomenological study of the influence of social support on well-being and performance in elite para swimmers

Authors Beth Aitchison, Alison B Rushton, Paul Martin, Andrew Soundy, Nicola R Heneghan

	**Sports medicine**	'she's [soft tissue therapist] the one that I just like rant to, she just listens'—	1
		'it's [soft tissue therapy] something that I really look forward to once a week'	5
		'the access we have to soft tissue treatment and physio treatment, are also super important […] access massage treatment more as you get closer to racing. The week before racing. Um. Or in like very very heavy training just to aid with recovery'	6
		'[physiotherapy is] just to kind of keep myself relatively loose during a heavy training period just so I don't get too tight and trying to prevent injuries then kind of heal them once I get it'	3
		'his [physiotherapist] role is basically to get my shoulder strong enough to swim […] keep me in the water as long as possible	1
		'you could go to the physio and say, "Look, this is recurring. What can I do to, to help it not happen?"'	4
		'he [physiotherapist] does give a bit of emotional support. He is one of, there's only a couple of people I trust to go to, but he can always spot when I'm having a bad day or, like he just knows straight away'	1
		'prevention rather than cure'	3
		'the more coaches are aware of what's proper pre-sport, post-sport, and uh, how to kind of prevent their swimmers from getting injured, the better	8
	**Financial support**	'I'm really grateful for the funding because if I didn't have it I wouldn't be able to swim'	2
		'I think having that support from UK Sport is, is extremely valuable. […]I feel extremely fortunate to be in this position'	8
		'obviously a massive, massive, um, weight off your shoulder and you can just, kind of, focus on, on your performance'	4
		'it [funding] allows athletes to be full time athletes; without the funding you're not a full-time athlete you're just someone that swims'	6
		'I think a lot of professional athletes don't realise how lucky we actually are to get that support'	5
		'the 'Bank of Mum''	2
		'sponsors'	4<br>5
		'[writing to] businesses asking for donations or sponsorship'	2
		'I wouldn't be able to live off what I get from British swimming, there's no way'	7
		'the most frustrating thing, is that we're all on funding, obviously, some are at different levels, but the rules should be the same for everyone and it's not'	1
		'there's no discrepancy between able-bodied and para'	8

		'you know like what times you're gonna have to swim to get such and such level of funding'	6
		it's very clear to everyone… like every year they set out um, either times or rankings that you need to do to qualify for a certain band of funding'	8
		'it might be quite useful if they could make like the criteria and everything a fair bit clearer	3
		'if you maintain world ranking your funding will remain the same, if you world ranking falls by X amount then funding will decrease by X amount. But not have that X amount be half. And if they could, if they could have a cap on how much funding could be decrease year on year, then that might be great'	6
**British para-swimming**	**'A happy swimmer is a fast swimmer'**	'when I'm training well, I'm happy; but also, when I'm happy, I'm training well, if that makes sense'	3
		'if you're happy where you are and if you've got a good programme, obviously it's got a good track record and good coach, um, then there's, there's no need to change it, really'	4
		'I think 'I'm very lucky that, like, I know what works best for me'	5
		'I'd never move because I really like that I have here'	2
	**'Best athlete that you can be'**	'the facilities in Manchester were amazing, the coaching was amazing, like we had everything we needed […] you basically couldn't ask for a better training environment'	6
		'anything I needed they [British Swimming] provided […] it was very kind of bespoke to what I needed'	8
		'their [British Swimming] support there is helping you be that best athlete that you can be'	5
		'I think in some areas we're very lucky because we've got people [support staff] who, in my opinion, are second to none'	8
		'we go through lifestyle and psych people like there's no tomorrow'	2
		'it would be nice if there was an EIS closer'	2
		'it'd be useful if they [support staff] were a little bit closer'	3
		'keeping people in the know all the time is, is something so important and having that trustworthy communication and respect, I think it's something that they can be a bit better on'	5
		'there's quite a lot of support there if you want it, but you have to, you have to get it. You have to chase it'	7
**The Podium Illusion**	**Performance support**	'The purpose of the S&C programme is to link into swimming, so it's to help the swimming, first and foremost'	4
		'He [S&C coach] relates the stuff in the water to land-based stuff'	5
		'The S&C work that you do translates to the swimming work that you do'	6

		'There are very few people who can get to high level sport without a strong S&C'	8
		'if I didn't have the equipment [used in S&C], I wouldn't be able, um, kind of build strength and power and really work on specific parts of my race'	3
		'I think a lot of people underestimate how important a start is, uh a dive start is. And I think the best way to develop that is one, practice, and two, strength [from S&C]'	8
		'Because it's so hard to translate, uh, strength on land to strength in the pool, uh, I think having an S&C coach who knows how to translate that strength into the water is incredibly important'	8
		'It's someone [psychologist] who will kind of support you and kind of look after you emotionally'	3
		'They gave me a lot of kind of advice, a lot of methods to help me um, when I was having a bit of a kind of plateau year'	3
		'Post-Paralympics or Olympics there's generally kind of a period where a lot of the athletes get a bit down, you don't really know what to do with yourself, you've had this goal built for so many years and now it's just finished, what do you do now? So I came back and was kind of a bit all over the place, didn't really know what I wanted to do'	6
		'[asking physiologist] should I eat this before I swim'	4
		'[asking physiologist] what's the best kind of warm up for this set?'	4
		'When I got back into the pool, I was then starting a different phase of training that I hadn't done before, and what we [swimmer, coach and physiologist] were doing was we were monitoring how effective the gym programme had been. And seeing how, seeing if that translated well into the water'	8
		'she's [nutritionist] been really helpful with me trying to expand my diet and like come up with better meal plans'	1
		'she [nutritionist] kind of gave me some more education on the diet I was wanting to do'	8
		'what I should be eating on a race day, I can use the nutritionist'	3
		'There's like a range that you should really be within when it comes to like skin folds and like power to weight. Um. So she [nutritionist] just helps me kind of get as close to that as possible'	6
		'the biomechanics team; they're involved in a lot of data analysis when we're at major meets. All the swimmers will be videoed and a race report will be created post-swim um so we can go back and um, evaluate that. It just has like the kind of bog-standard stuff that you would expect like stroke rates and splits, rotation time at the wall'	6

The Podium Illusion: A phenomenological study of the influence of social support on well-being and performance in elite para swimmers

Authors Beth Aitchison, Alison B Rushton, Paul Martin, Andrew Soundy, Nicola R Heneghan

		'using kind of race analysts to work on technical parts, so, like, stroke rate'	3
		'Every para competition, uh, pretty much and breaks down all your splits, stroke rates and things like that. So it just gives you a wider understanding of how you, how you swim the race, and what's the best way to swim the race'	8
		'they [home programme liaison] communicate between the Performance Centre and people in their home programmes'	3
		'he's [home programme liaison] kind of like a bridge between us [athlete and coach] and the rest of British Swimming'	8
		'they're [home programme liaison] a good person to talk to as well if you want anything, if you want to kind of say something'	3
	**Personal support**	'[friends and family] obviously know you best and, um, want the best for you, like away from the sport as well'	4
		'you couldn't do it on your own'	4
		'My mum and my friends keep me in the right head space to train, especially when I'm going through like injuries'	1
		'I'm so lucky that I've got that support system around where I can talk and it, it helps me, like, really think about things and it's huge, yeah, support system to have, to have them in place'	5
		'We're [mum and athlete] really close. I tell her like everything. Um. And I think if there was ever a time where I felt stressed or vulnerable or upset she would probably be the first person I spoke to'	6
		'Really kind of understanding of like, "These are all the commitments that [athlete name]." They [friends] understand if I can't make something, if I can't do something then it's because of swimming'	3
		'[my partner] doesn't know nothing about swimming and he's just like, "Oh, yeah, you're gonna do so well!"	2
		'[after a race] I'm already kind of looking at my times and kind of picking apart races and things like that, and my coaches also do that as well. So, to have like a third party [parents] doing that, it'd just be too much'	3
		'I think it's nice having a performance lifestyle advisor because they can really help you put something in place, they can help you find a bit of a career, so you never feel like, you never feel lost'	3
		'To have someone [PL] there to kind of reassure you, and just kind of provide you with all the information, and just say it's okay'	8
		'He [agent] knows when I can do stuff, when I can't due to competitions. So, again, it frees me of one less job to do, so I can actually go out there and, uh, do my training and perform, so I'm not having to think about replying to all these emails'	5

		‘He’ll [agent] come back and say, “Have you done this? Have you done that? Have you done that?” So he’ll keep me on, on track, make sure I’ve done everything’	7
		I look back on it now and have really, really fond memories. And, um, yeah, it, it was just great fun and it was, it was almost a bit of a comfort having somebody [sibling] there, I think.	4
